# Machine Learning-Based Etiologic Subtyping of Ischemic Stroke Using Circulating Exosomal microRNAs

**DOI:** 10.3390/ijms25126761

**Published:** 2024-06-20

**Authors:** Ji Hoon Bang, Eun Hee Kim, Hyung Jun Kim, Jong-Won Chung, Woo-Keun Seo, Gyeong-Moon Kim, Dong-Ho Lee, Heewon Kim, Oh Young Bang

**Affiliations:** 1 Global School of Media, College of IT, Soongsil University, Seoul 06978, Republic of Korea; qkddlfkdrp@gmail.com; 2 S&E Bio, Inc., Seoul 05855, Republic of Korea; 3 Department of Neurology, Samsung Medical Center, Sungkyunkwan University School of Medicine, Seoul 06351, Republic of Korea; 4 Calth, Inc., Seongnam-si 13449, Republic of Korea; 5 Department of Health Sciences and Technology, Samsung Advanced Institute for Health Sciences and Technology (SAIHST), Sungkyunkwan University, Seoul 06351, Republic of Korea

**Keywords:** ischemic stroke, subtype, etiology, extracellular vesicle, microRNAs, machine learning

## Abstract

Ischemic stroke is a major cause of mortality worldwide. Proper etiological subtyping of ischemic stroke is crucial for tailoring treatment strategies. This study explored the utility of circulating microRNAs encapsulated in extracellular vesicles (EV-miRNAs) to distinguish the following ischemic stroke subtypes: large artery atherosclerosis (LAA), cardioembolic stroke (CES), and small artery occlusion (SAO). Using next-generation sequencing (NGS) and machine-learning techniques, we identified differentially expressed miRNAs (DEMs) associated with each subtype. Through patient selection and diagnostic evaluation, a cohort of 70 patients with acute ischemic stroke was classified: 24 in the LAA group, 24 in the SAO group, and 22 in the CES group. Our findings revealed distinct EV-miRNA profiles among the groups, suggesting their potential as diagnostic markers. Machine-learning models, particularly logistic regression models, exhibited a high diagnostic accuracy of 92% for subtype discrimination. The collective influence of multiple miRNAs was more crucial than that of individual miRNAs. Additionally, bioinformatics analyses have elucidated the functional implications of DEMs in stroke pathophysiology, offering insights into the underlying mechanisms. Despite limitations like sample size constraints and retrospective design, our study underscores the promise of EV-miRNAs coupled with machine learning for ischemic stroke subtype classification. Further investigations are warranted to validate the clinical utility of the identified EV-miRNA biomarkers in stroke patients.

## 1. Introduction

Stroke is a devastating disease that is prevalent worldwide. Ischemic stroke, which is caused by arterial blockage of the brain, constitutes the majority (80%) of stroke cases. Ischemic stroke can be categorized into distinct etiological subtypes, including large artery atherosclerosis (LAA; narrowing or blockage of a major artery by plaque), cardioembolic stroke (CES; migration of blood clots in the heart formed by atrial fibrillation [AF], heart valve disorders, and cardiac thrombus), and small artery occlusion (SAO; occlusion of small perforating arteries within the brain, often associated with hypertension) [1]. Determining the underlying cause of ischemic stroke is crucial, as tailored prevention strategies hinge upon accurately identifying these subtypes, and the etiology of recurrent strokes typically mirrors that of the initial event [2,3]. For instance, management approaches differ significantly, with recommendations such as dual antiplatelet therapy and high-dose statins for LAA-related stroke, anticoagulant therapy for CES, and risk factor control for SAO. Given the necessity of comprehensive evaluations involving brain, vascular, and cardiac assessments [2], the existence of a simple blood test capable of elucidating stroke subtypes would greatly benefit clinical practice.

MicroRNAs (miRNAs) are promising diagnostic candidates. These small non-coding RNA molecules regulate gene expression via post-transcriptional regulation and exhibit high specificity and sensitivity as diagnostic and prognostic markers for various diseases [4]. Unlike genome-wide association studies (GWAS), circulating miRNAs are dynamic biomarkers that reflect real-time changes in stroke pathophysiology. Notably, circulating miRNAs encapsulated in extracellular vesicles (EV-miRNAs) remain relatively stable in biological fluids [5,6]. Recent studies have highlighted altered profiles of circulating EV-miRNAs in patients with acute ischemic stroke due to SAO or cancer-related coagulopathy, paving the way for their consideration as diagnostic markers and therapeutic targets [7,8,9].

In this study, we hypothesized that measuring EV-miRNA levels could aid in predicting etiological subtypes in patients with acute ischemic stroke. Using next-generation sequencing (NGS) and machine-learning (ML) techniques, we assessed the diagnostic accuracy of EV-miRNA profiles in distinguishing between LAA, SAO, and CES. Bioinformatic analyses were employed to elucidate the potential functional roles of differentially expressed miRNAs (DEMs) in stroke subtypes.

## 2. Results

This section is divided into several subheadings. A concise and precise description of the experimental results, their interpretation, and experimental conclusions are provided below.

### 2.1. Clinical Characteristics of Patients with Three Stroke Subtypes

This study recruited 70 patients with acute ischemic stroke. After comprehensive workups for stroke etiologies, 24 were classified as the LAA group, 24 as the SAO group, and 22 as the CES group. Patent’s characteristics, including the prevalence of vascular risk factors, were not different among the groups (Table 1).

### 2.2. Characteristics of Circulating Extracellular Vesicles

**Figure 1:**
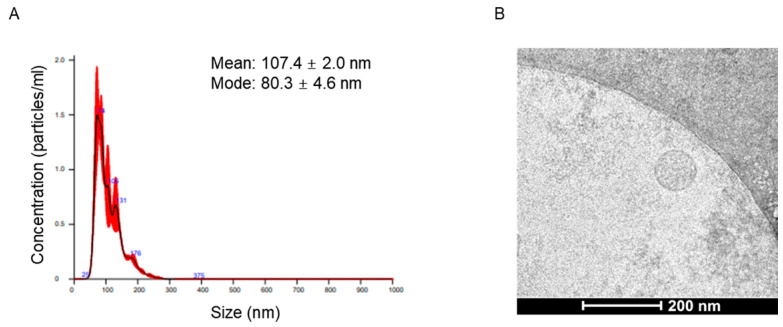
Characteristics of plasma extracellular vesicles. (**A**) Size distribution of EVs, as determined by NanoSight tracking analysis. (**B**) Size and lipid double layers of purified EVs, as determined by electron microscopy.

Cryo-TEM revealed typical EV features, and EV marker positivity was confirmed (Figure 1). Most EVs had a round shape with an electron-dense structure, and the mode diameters of EVs were 80.3 ± 4.6 nm. The circulating EVs were positive for EV markers such as CD63 and TSG101 and negative for calnexin, an EV negative marker. 

### 2.3. Comparison of miRNA Profiling of sEV-Derived miRNAs among Stroke Subtypes

**Figure 2:**
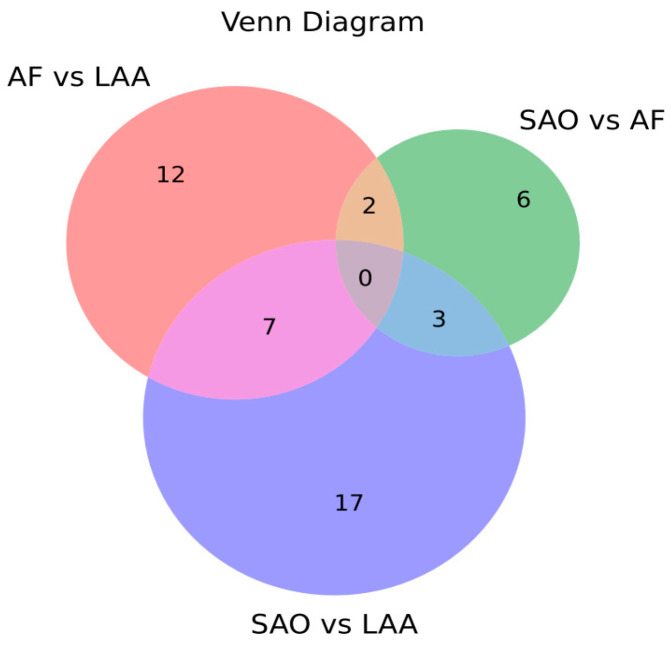
Venn diagram depicting the microRNAs identified in the three stroke subtypes using next-generation sequencing.

Small RNA sequencing revealed the differential expression of EV-miRNAs among stroke subtypes with several miRNAs showing subtype-specific alterations (Figure 2 and Supplementary Table S2). 

#### 2.3.1. Performance Comparisons of ML Models 

**Figure 3:**
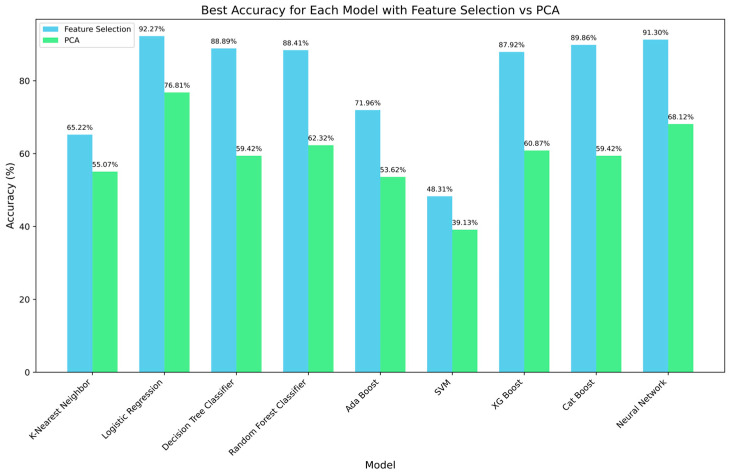
Best accuracy of machine-learning models: feature selection vs. principal component analysis (PCA). This figure provides a side-by-side comparison of the best accuracy achieved by a range of machine-learning models using two-dimensionality-reduction techniques: feature selection (blue) and PCA (green). The feature selection using the chi-square test and PCA are utilized by “SelectKBest” and “PCA” in the scikit-learn library. The reported accuracy of each model was the best when the number of features for feature selection and the number of principal components for PCA were varied. The accuracies are displayed as percentages, demonstrating the superior performance of feature selection over PCA in most models. Logistic regression, in particular, exhibits a notable increase in accuracy with feature selection compared with PCA, highlighting the importance of appropriate feature engineering in model optimization.

We compared the performances of feature selection and principal component analysis (PCA) using various ML methods. PCA could not provide clear class separation (Supplementary Figure S1), and feature selection outperformed PCA in all ML models (Figure 3). When comparing the performance across the ML models, logistic regression demonstrated the highest accuracy, followed by neural networks. These findings suggest that feature selection captures the important characteristics of the data more effectively, highlighting its significance as a methodology for optimizing model prediction performance.

#### 2.3.2. Impact of Feature Selection 

**Figure 4:**
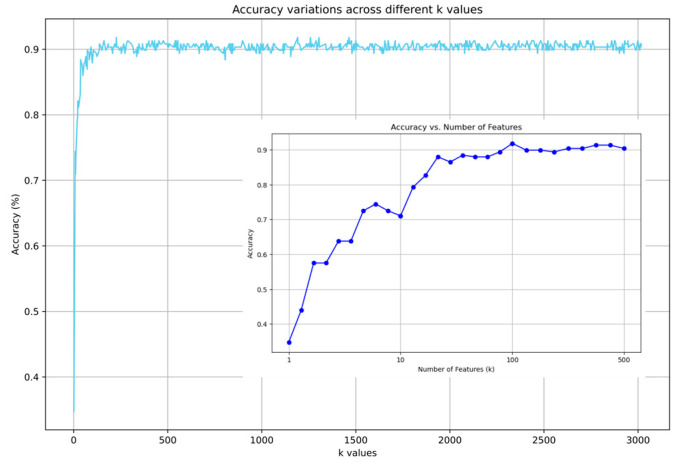
Logistic regression accuracy when varying the number of features (*k*) selected by chi-square test. This graph illustrates the accuracy of the logistic regression models as a function of the number of features (*k*) selected using the chi-square test from the SelectKBest method in scikit-learn. The main plot displays the accuracy variations across k values ranging from 1 to 3017. The inset provides a detailed view, showing a significant improvement in the accuracy up to 100, where it peaks above 90%, followed by fluctuations at higher k values. The highest reported accuracy was 92.27% at *k* = 1251, highlighting the optimal feature subset size for differentiating ischemic stroke subtypes.

**Figure 5:**
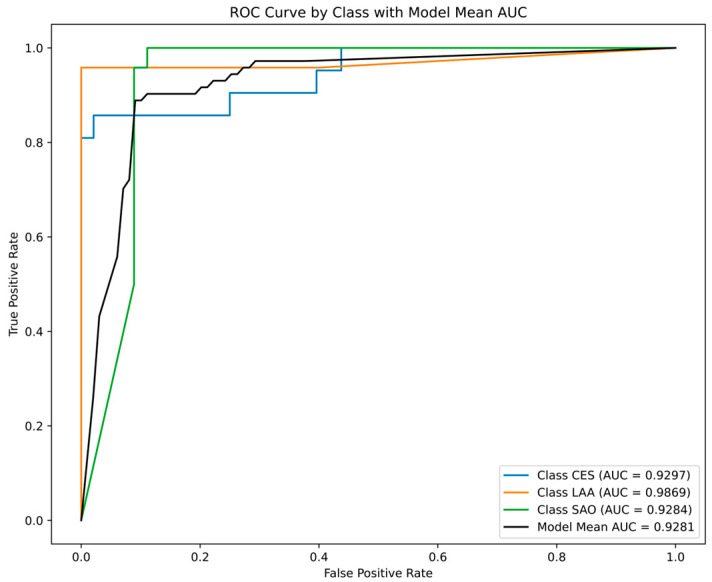
Receiver operating characteristic (ROC) curves for stroke classification. The graph shows the results of the ROC curves for the three different classes of stroke diagnoses: cardioembolic stroke (CES), large artery atherosclerosis (LAA), and small artery occlusion (SAO). The area under the curve (AUC) for each class is a measure of the model’s ability to discriminate between positive and negative cases.

The number of features (*k*, from 1 to 3017) significantly influenced the accuracy of the logistic regression. The accuracy significantly improved from *k* = 1, where it was <40%, to *k* = 100, where it exceeded 90%, then fluctuated for *k* > 100 (Figure 4). This phenomenon indicates that not all features represent the information for ischemic stroke subtypes and that an effective number of features exist for model performance. A setting of *k* = 1251 reports the best accuracy of 92.27%. In all experimental settings, logistic regression with 1251 selected features achieved the highest accuracy of 92.27%, with correct predictions for each class of 85.71% for CES, 98.61% for LAA, and 91.67% for SAO. Figure 5 illustrates the receiver operating characteristic (ROC) curves of each class and the area under the ROC curve (AUC) of 0.9274 for CES, 0.9870 for LAA, and 0.9298 for SAO.

#### 2.3.3. Impact of Clinical Information on EV-microRNA Prediction Models

We evaluated the impact of integrating clinical information (age, sex, and vascular risk factors) with miRNA expression profiles on the accuracy of stroke subtype classification. Clinical data integration minimally affected the predictive performance (Supplementary Figure S2).

### 2.4. Underlying Mechanisms of EV-miRNAs for Each Stroke Subtype

#### 2.4.1. Feature Importance Analysis

Subsequently, we visualized and quantified the top 10 features that contributed positively to the discrimination of each class and the bottom 10 features that adversely affected the discrimination process (Supplementary Table S2). 

#### 2.4.2. Bioinformatics Analysis

**Figure 6:**
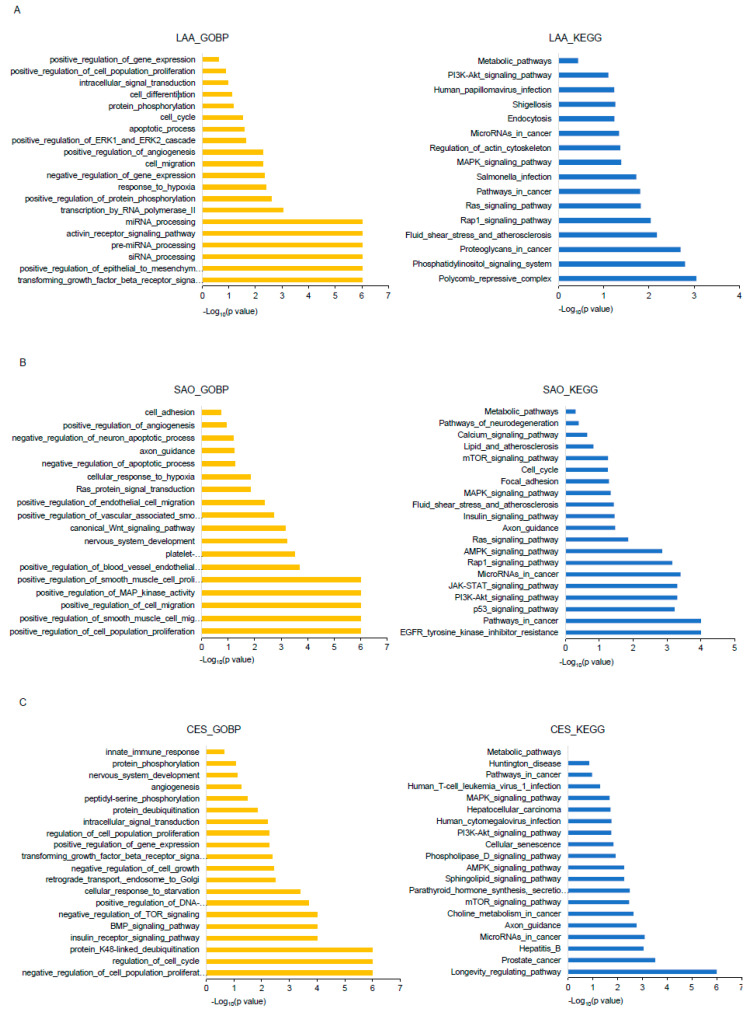
Gene Ontology (GO) and Kyoto Encyclopedia of Genes and Genomes (KEGG) pathway enrichment analysis of DEMs. GO of biological processes and KEGG pathways for (**A**) large artery atherosclerosis (LAA), (**B**) small artery occlusion (SAO), and (**C**) cardioembolic stroke (CES), presented using the 10 most relevant terms for the target genes of miRNAs (top 10 contributing features of Supplementary Table S2) enriched in EVs.

The top 10 DEMs were analyzed for their potential functional roles in stroke subtypes. We detected the function of miRNAs expressed in the target genes using the Gene Ontology (GO) and Kyoto Encyclopedia of Genes and Genomes (KEGG) pathway analyses. The miRNAs enriched in the EVs of the LAA-induced stroke group interacted with the Ras signaling pathway, MAPK signaling pathway, fluid shear stress and atherosclerosis, and transforming growth factor-β receptor signaling pathway through target genes such as VEGFA, TGFBR3, or ITGB3 (Figure 6A). The enriched miRNAs in the EVs of the SAO group interacted with target genes such as FGF2, PIK3R1, and PDGFRB and were linked to the Ras signaling pathway, Janus kinase/signal transducer and activator of transcription (JAK/STAT) signaling pathway, platelet-derived growth factor receptor signaling pathway, Wnt signaling pathway, and angiogenesis (Figure 6B). The miRNAs enriched in the EVs of the CES group were found to be associated with the calcium signaling pathway, AMPK signaling pathway, MAPK signaling pathway, or transforming growth factor-β receptor signaling pathway through TP53 and IGF1R (Figure 6C).

## 3. Discussion

A typical method for identifying specific miRNAs and their cutoff values for diagnosing a certain disease involves the selection of candidate miRNAs based on a literature review, qRT-PCR expression levels, and biological functions. In the present study, we failed to select representative DEMs for validation using qRT-PCR. Our findings indicated that when fewer than five miRNAs were measured, the diagnostic rate ranged from 35% to 75% for 1–10 features. With more than 100 features measured, the accuracy reached 89%, and with 1251 features analyzed, the accuracy was 92%, demonstrating a more precise prediction rate when a larger number of miRNAs were measured. Several miRNAs, not just a single miRNA, can orchestrate fundamental biological processes in stroke development. According to the miRbase database, there are 4,475,477 miRNA-target gene interactions between 3012 miRNAs and 17,387 target genes, and these counts are rapidly growing. Therefore, a single miRNA may not be important, and the miRNA profile may be more important. Although one miRNA can regulate hundreds of genes, its effect on each gene may not be sufficient, and several miRNAs may simultaneously suppress one gene. In addition, the regulatory loops that govern miRNA-miRNA and miRNA-mRNA interactions are currently under investigation [10]. Our results showed that the advantages of using ML include (a) handling high-dimensional data allowing the simultaneous analysis of a large number of features, which is crucial given the complex and vast dataset derived from NGS of EV-miRNAs, (b) identifying pattern recognition and feature selection within biological data that may not be apparent through traditional statistical methods, (c) integration of multiple data types, such as miRNA profiles, clinical information, and neuroimaging data, although the integration of clinical data minimally affected predictive performance in this study, and (d) high diagnostic accuracy in classifying. Zhang et al. recently reported a single-EV approach for miRNA profiling using ML in cancer diagnosis and classification [11].

Recently, the integration of ML into medical diagnostics has marked a significant milestone in the evolution of healthcare and has been increasingly applied to stroke patients [12,13]. These techniques help to identify meaningful patterns within complex biological data. In the present study, we used NGS and ML techniques to demonstrate the utility of EV-miRNA profiling for predicting stroke subtypes. ML techniques can analyze large datasets of miRNA expression levels with high precision and accuracy, potentially identifying complex patterns within the data and subtle patterns/associations that may not be apparent to researchers. The ML algorithms used in our study have managed the high dimensionality of miRNA data and are capable of identifying non-linear and subtle patterns that may signify important biological interactions relevant to stroke. However, the interpretability of such models remains a critical hurdle because the “black box” nature of these algorithms often makes clinical translation and trust in decision-making difficult [14]. There is a potential for ML to misclassify owing to underlying biases in the training data or overfitting, especially when dealing with small data sizes. Our results demonstrate that feature selection outperforms PCA. Given the importance of interpretability, preservation of original features, and semantic importance, feature selection is more suitable than PCA [15].

Our results showed that a significant number of patients with one subtype were classified as having other subtypes based on DEMs. There are several possible explanations for this observation. First, patients with acute ischemic stroke may have more than one stroke pathomechanism [16]. For example, patients with SAO may have subclinical features of CES (i.e., left atriopathy, as measured by the left atrial volume index [LAVI]). However, in this study, there was no significant difference in LAVI between CES predicted as SAO and CES predicted as CES (81.1 ± 39.3 and 72.9 ± 42.8, *p* = 0.661). Similarly, there was no difference in LAVI between SAO predicted as SAO and SAO predicted as CES (38.3 ± 17.4 and 40.2 ± 6.9, *p* = 0.799). Leukoaraiosis and cerebral microbleeds were frequently observed in patients with SAO but were rare in patients with CES, regardless of the miRNA profiles. Therefore, it is unlikely that the similarity in miRNA profiles between SAO and CES is caused by the presence or severity of the subclinical features of the stroke subtypes. Second, DEMs could represent genetic factors for the risk factors of stroke as well as those for causative factors. Stroke subtypes share common risk factors (such as hypertension and smoking) and genetic predispositions related to blood clotting disorders or vascular health. GWASs have identified several stroke subtype-specific nucleotide polymorphisms and polygenic risk scores, including genetic factors associated with vascular risk factors [17,18,19].

In the present study, we evaluated the expression profile of miRNAs encapsulated in EVs because EVs are relatively stable in the bloodstream, whereas miRNAs have a short half-life in circulation [6]. However, discrepancies in EV-miRNA and EV proteomic patterns are frequent among studies, which may be partly due to differences in the sample type from which EVs are derived (i.e., plasma or serum) and methodological differences in EV isolation, miRNA profiling, and expression normalization [20,21,22,23]. It is important to develop reproducible methods for isolating EVs from biological samples with high yield and purity. Different DEMs were associated with SAO [7,8]. Van Kralingen et al. isolated EVs using a total exosome isolation reagent and reported that circulating EV-miRNA-17 family members were increased in both patients with SAO and an animal model of SAO [7]. Otero-Ortega et al. used the ExoQuick Ultra EV precipitation method and showed that the expression of miR-15a, miR-424, miR-100, and miR-339 was higher in patients with SAO than in those with cortical infarcts [8].

Furthermore, we elucidated the underlying mechanisms of EV-miRNAs in stroke subtypes using bioinformatics analyses. Enrichment analyses identified distinct pathways and biological processes associated with differentially expressed DEMs in each subtype. Notably, EV-miRNAs enriched in LAA were linked to pathways involved in atherosclerosis and vascular remodeling, whereas those in SAO were associated with angiogenesis and inflammation. CES-associated EV-miRNAs were associated with calcium signaling and stress response pathways.

This study had several limitations. First, the sample size of the patient cohort was relatively small because NGS was performed on all patients. Given the limited sample size, retrospective design, and data from a single center in Asia, caution should be exercised when generalizing the results. In addition, in this study we focused on the differentiation of three major stroke subtypes; however, approximately one fourth of strokes are cryptogenic. Secondly, several cell types in the brain and circulation release EVs into the blood during stroke; however, the source of EVs/miRNAs were not evaluated in this study. Further studies are required to evaluate the sources of EVs and EV-miRNAs. Third, owing to the small number of patients, presenting more standardized results was difficult; consequently, our study’s outcomes varied with different random states, yielding different numbers for the most efficient feature selection and models. In other words, there is a lack of generalizability, and it appears that additional data and research are necessary to address this issue, which could lead to significant improvements in the accuracy of the second-best performing model, the neural network, owing to its characteristics (Supplementary Figure S3) [24]. Finally, the clinical and imaging characteristics of the patients, including infarct size and medications/acute interventions, were not analyzed because of the small cohort size. In addition, future studies should use multiple biomarkers (including EV proteins) to determine whether the use of a combination of biomarkers can improve the prediction of stroke subtypes because the contribution of EVs to stroke subtypes could be complex.

In conclusion, EV-miRNA profiles exhibit subtype-specific alterations in ischemic stroke, offering promise as diagnostic markers when ML techniques are applied to analyze a large NGS dataset. Notably, the collective influence of multiple miRNAs was more crucial than that of individual miRNA. Further prospective studies are warranted to validate representative EV-miRNAs as potential biomarkers of stroke subtypes.

## 4. Materials and Methods

### 4.1. Patient Selection

We prospectively studied consecutive patients with acute ischemic stroke admitted to a university medical center between April 2016 and May 2018. Potential participants were defined as patients who experienced focal or lateralizing symptoms within 7 days of symptom onset and showed relevant lesions on diffusion-weighted imaging (DWI). Clinical information, including age, sex, and vascular risk factors was also collected. All patients underwent diagnostic testing including routine blood tests, electrocardiography, at least 24 h of cardiac telemetry, and echocardiography. Vascular imaging was conducted using 3D time-of-flight magnetic resonance angiography (MRA) for the intracranial arterial system and contrast-enhanced MRA including the extracranial internal carotid and vertebral arteries for the extracranial arterial system using 3.0-tesla MRI scanners. Additionally, some patients underwent computed tomography (CT) angiography, ultrasound, or high-resolution vessel wall MRI to improve the diagnostic accuracy for the detection of significant stenosis and differentiation between atherosclerotic and non-atherosclerotic stenosis (e.g., arterial dissection and moyamoya disease) [25].

Patients were grouped by the presumed stroke mechanism as (1) the LAA group, patients with a significant (≥50%) stenosis in the relevant artery and no proximal source of embolism, (2) the SAO group, patients with a small (<2 cm) subcortical infarction without evidence of significant stenosis in the relevant artery, and (3) the CES group, patients with AF and large (≥2 cm) or cortical lesions on DWI without significant (≥50%) occlusive disease on the proximal relevant artery [1]. The diagnosis of AF was based on electrocardiographic findings and/or 24 h Holter monitoring during hospitalization, as assessed by a cardiologist or using data from medical history with electrocardiography-documented AF. We excluded patients with (1) no presumed stroke mechanisms, (2) two or more stroke mechanisms, (3) other stroke mechanisms (coagulopathy, vasculitis, moyamoya disease, artery dissection, and others), and (4) incomplete evaluations.

Our definitions of vascular risk factors were as follows: (1) hypertension was deemed present when the patient had been undergoing treatment with antihypertensive agents or had a blood pressure of either ≥140 mmHg systolic or ≥90 mmHg diastolic on at least two occasions after the acute phase of their ischemic stroke. (2) Diabetes mellitus was deemed present when the patient had been receiving medication for diabetes and had an elevated fasting glucose level ≥126 mg/dL or hemoglobin A1c level >6.5%. (3) Dyslipidemia was considered present if the patient had been taking lipid-lowering agents or had a total cholesterol level of >240 mg/dL, triglyceride level of >200 mg/dL, or low-density lipoprotein cholesterol level of >160 mg/dL. (4) Current smokers were defined as those who had smoked more than 100 cigarettes in their lifetime and had smoked within the last 28 days. (5) Alcohol consumption was assessed in all patients, using a structured questionnaire about alcohol intake [26]. Average daily alcohol consumption was divided into three categories: no drink, light–moderate (1–4 drinks per day, 1 drink = 10 g ethanol), and heavy (5 or more drinks per day).

### 4.2. Isolation and Characterization of EVs

Peripheral blood was obtained during the acute period (within seven days of symptom onset). EVs were isolated from citrate plasma samples and characterized based on morphology, size distribution, and surface markers, following recommended guidelines [27,28]. Citrated whole blood samples were centrifuged at 2000× *g* for 15 min to obtain citrate plasma samples, which were stored at −80 °C until further analysis. Citrate plasma was centrifuged at 1000× *g* for 10 min at 4 °C. The supernatant was centrifuged at 100,000× *g* for 1 h at 4 °C using an Optima TLX ultracentrifuge (Beckman Coulter, Brea, CA, USA) and a TLA120.2 rotor to isolate EVs. The final pellet containing EVs was resuspended in 100 μL of filtered phosphate buffered saline (PBS).

EVs were pre-diluted in vesicle-free water and their concentration and size distribution were characterized using a NanoSight NS300 system (Malvern, Worcestershire, UK). The mean particle size and concentration (particles/mL) were calculated by integrating the data from three individual measurements. Direct visualization of EVs was performed using a cryo-transmission electron microscopy (TEM). Carbon grids (Quantifoil, R1.2/1.3, 200 mesh, EMS; Hatfield, PA, USA) were made hydrophilic with glow-discharge using a Pelco EasiGlow system (TED PELLA, Redding, CA, USA). An aliquot (4 μL) of samples was placed on the carbon side of the EM grid and blotted for 1.5 s with 100% humidity at 4 °C. The samples were plunge-frozen in precooled liquid ethane using a Vitrobot Mark IV (FEI, Hillsboro, OR, USA). The samples were analyzed using a Talos L120C cryo-electron microscope (FEI) at 120 kV.

### 4.3. RNA Isolation

The total RNA of the EVs was extracted using the miRNeasy Serum/Plasma Kit (Qiagen, Hilden, Germany) according to the manufacturer’s instructions. Extracted RNA was eluted in 14 μL RNase-free water and stored at −80 °C. RNA concentration was quantified using a NanoDrop 1000 spectrophotometer (NanoDrop, Wilmington, DE, USA).

### 4.4. miRNA Profiling

The expression profiles of miRNAs encapsulated in plasma EVs were evaluated using small RNA sequencing. Libraries were prepared for 50 bp single-end sequencing using the NEXTflex Small RNA-Seq Kit (Bioo Scientific Corp, Autstin, TX, USA). Specifically, small RNA molecules were isolated from 1 μg of total RNA via the adapter ligation. The isolated small RNAs were synthesized as single-stranded cDNAs through RT (reverse transcription) priming. Using this as a template for second strand synthesis, double-stranded cDNA was prepared by polymerase chain reaction (PCR). Fragments of approximately 150 bp were extracted for sequencing by size selection by gel electrophoresis. The quality of these cDNA libraries was evaluated using an Agilent 2100 BioAnalyzer (Agilent, CA, USA) followed by quantification using a KAPA library quantification kit (Kapa Biosystems, MA, USA) according to the manufacturer’s protocol. Following cluster amplification of the denatured templates, sequencing was progressed as single-end (50 bp) using Illumina NovaSeq 6000 150PE (Illumina, San Diego, CA, USA). Low quality bases or reads were trimmed or filtered using the following criteria: bases with a quality score of less than 20 and read length with below 17 bp were subject to trimming. The entire process was performed using the Cutadapt tool [29]. Filtered reads were mapped to the reference genome of the related species using the aligner Bowtie [30], followed by variant calling in the seed region of the miRNA being performed. For the expression estimation, mirdeep2 tool was used [31]. The variant calling was performed using the GATK to search for variants in the miRNA seed region. The dcov option was set to 1000 as the maximum depth threshold in this region [32]. miRNA expression levels were measured with mirdeep2 using the gene annotation database of the species along with hairpin and mature miRNA sequence information, which can be extracted from miBase [31,33]. All parameters were set to their default values.

### 4.5. Data Analysis Step

We used Python with the scikit-learn 1.2.0 library for all analyses through the following steps: (1) feature selection, (2) data pre-processing and partitioning, (3) ML analysis for predictive classification modeling, and (4) ranking variable importance.

(1) Feature selection: Given that not all miRNA features affect stroke, we utilized the chi-square test to select influential features related to ischemic stroke [15]. The chi-square test evaluates the independence between the given features and class labels using the chi-square statistic. A higher value of the statistic indicates a stronger relationship between the features and classes. We selected the top *k*-related features in the training dataset using “SelectKBest” from the scikit-learn library. For comparison with feature selection, we employed PCA, which reduces the dimensions of high-dimensional data while preserving the information. The PCA transforms the given features into a new set of features, called principal components, which are less related to each other [34]. We utilize the *k* principal components in the training dataset using “PCA” from the scikit-learn library.

(2) Data pre-processing and partitioning: To prepare the raw data for ML model construction, categorical variables (CES, SAO, and LAA) were encoded as variables with three elements using one-hot encoding. The remaining numerical features were scaled using “MinMaxScaler” from the scikit-learn library, which transforms them to a range between 0 and 1 [35]. We utilized 3-fold cross-validation to separate train and test data, employing “cross_validate” from the scikit-learn library. This approach improves the accuracy of assessment of the model performance by averaging the test scores from each fold.

(3) ML analysis for predictive classification modeling: To predict 3 classes of brain stroke, we used various classification models that were provided in the scikit-learn package for uniform execution: ”Logistic Regression”, ”KNearest Neighbor”, ”Decision Tree Classifier”, ”Random Forest Classifier”, ”Ada Boost”, ”SVM”, ”XG Boost”, ”Cat Boost”, and ”Neural Network” (Supplementary Table S1) [36]. For the model selection of each ML algorithm (a crucial step in reducing the model’s error value) we utilized scikit-learn’s hyperparameter tuning utilities, applied them to the down-sampled training data, and set the model parameters accordingly. The performance of the models was evaluated by examining and calculating the AUC, accuracy, precision, sensitivity, specificity, and F1-score. AUC, a widely used performance metric for classification problems, is generally considered good between 0.8 and 0.9, with values above 0.9 considered excellent. A higher F1-score indicates better correct identification of classes, signifying fewer false positives and false negatives. In this study, the accuracy, precision, sensitivity, specificity, and F1-score were evaluated using a confusion matrix.

(4) ML analysis for predictive classification modeling: The ML models were analyzed by evaluating feature importance through correlation and contribution analyses. All the models assessed the importance of each feature by measuring its impact on the outcome. This analysis incorporated the weight coefficients in the logistic regression and node split-importance in the linear models. In the scikit-learn package, we employ “coef_” for linear models to determine feature importance evaluation.

### 4.6. Bioinformatics Analysis of miRNAs

For DE miRNA analysis, miRNA level count data were generated using mirdeep2 [31]. Based on the calculated read count data, DE miRNAs were identified using the R package TCC, which applies robust normalization strategies to compare tag count data [37]. The normalization factors were calculated using the iterative DEGES/edgeR method. The Q-value was calculated based on the *p*-value using the p-adjusted function of the R package with default parameter settings. The DE miRNAs were identified to correct errors caused by multiple tests based on a q-value threshold of < 0.05. We detected the function of selected differentially expressed miRNAs in target genes using KEGG and GO pathway analyses. GO and KEGG pathway enrichment analyses were performed using the miRWalk2.0 web-based tool (http://mirwalk.umm.uniheidelberg.de/ accessed on 17 January 2024). Potential miRNA targets were predicted using Targetscan v8.0 (https://www.targetscan.org, accessed on 17 January 2024) and the mirDB database (https://mirdb.org/, accessed on 17 January 2024).

### 4.7. Statistical Analysis

Differences in discrete variables between the groups were evaluated using the χ^2^, Fisher’s exact, or Mann–Whitney U test. Differences in continuous variables were evaluated using one-way analysis of variance (ANOVA), Kruskal–Wallis test, or *t*-test. Dunnett’s method was used for multiple comparisons. ROC curves were used to compare the discriminatory power of miRNA features for differentiating of stroke subtypes. We assessed the discrimination power by calculating the AUC. An area of 1 implies that the test has perfect sensitivity and specificity, whereas an area of 0.5 implies that the model’s predictions are no better than chance. The best model was defined as the one with the largest ROC curve. A two-tailed *p* value of <0.05 was considered statistically significant. All statistical analyses were performed using commercially available software (SPSS Statistics version 24.0, IBM Corp., Armonk, NY, USA).

## Figures and Tables

**Table 1:** Patients’ characteristics.

	LAA	SAO	CES	*p*-Value
Age	72.5 ± 8.5	67.0 ± 11.8	73.3 ± 10.0	0.077
Male sex	20 (83.3%)	17 (70.8%)	17 (77.3%)	0.588
Risk factor				
Hypertension	19 (79.2%)	19 (79.2%)	16 (72.7%)	0.837
Diabetes	11 (45.8%)	9 (37.5%)	7 (31.8%)	0.616
Dyslipidemia	13 (54.2%)	13 (54.2%)	9 (40.9%)	0.588
Smoking				0.300
Never	9 (37.5%)	12 (50.0%)	12 (54.5%)	
Ex smoker	7 (29.2%)	5 (20.8%)	8 (36.4%)	
Current smoker	8 (33.3%)	7 (29.2%)	2 (9.1%)	
Alcohol				0.607
None	14 (58.3%)	18 (75.0%)	14 (63.6%)	
Light–moderate	5 (20.9%)	6 (25.0%)	5 (22.6%)	
Heavy	5 (20.9%)	-	3 (13.5%)	

## Data Availability

All original data supporting reported results can be made available upon request.

## Supplementary Materials

The following supporting information can be downloaded at: https://www.mdpi.com/article/10.3390/ijms25126761/s1.
