# Supplementary material for: Machine Learning-Based Etiologic Subtyping of Ischemic Stroke Using Circulating Exosomal microRNAs
Source: Int J Mol Sci. 2024 Jun 20;25(12):6761. doi: 10.3390/ijms25126761 (PMC11203849; doi:10.3390/ijms25126761)
Supplement: Supplementary file 1 [file ijms-25-06761-s001.zip › ijms-3030884-supplementary.pdf]

Supplementary Information

Table S1. ML algorithms used in the current study

Model	Description	Hyperparameter used in skitlearn
**Logistic Regression**	Logistic Regression serves as a straightforward yet powerful linear classification algorithm, particularly adept at handling binary and multi-class classification challenges. This method establishes a relationship between independent variables and the probability of a specific outcome, employing the logistic function. By modeling a logistic curve, it estimates the likelihood of an instance belonging to a given class. Renowned for its simplicity and interpretability, logistic regression proves efficient in scenarios where linearly separable data is at play	penalty=12, C=1.0
**Ada Boost**	AdaBoost is also an ensemble method like random forest. The core principle of AdaBoost is to fit a sequence of “weak learners” (i.e., models that are only slightly better than random guessing) to repeatedly modified data. All predictions are then combined through a weighted majority vote (or sum) to generate the final prediction.	n_estimators=50, learning_rate=1.0, random_state=11
**Random Forest Classifier**	Random forest is a tree-based ensemble method that utilizes parallel decision trees built on subsets of the data to develop an optimal predictive model. Each tree in the random forest casts a vote based on its prediction, and the classification with the most votes becomes the overall model’s prediction	n_estimators=100, criterion='gini', random_state=11
**Cat Boost**	CatBoost steps into the spotlight as a gradient boosting algorithm tailor-made for navigating the complexities of categorical features. Its prowess lies in a unique blend of ordered boosting and an innovative categorical feature encoding technique. This algorithm shines by automating the treatment of missing values and minimizing the need for exhaustive pre-processing. Recognized for its robust performance, efficiency, and user-friendly nature, CatBoost emerges as a top choice for both classification and regression tasks.	iterations=500, learning_rate=0.03, logging_level='Silent', random_state=11
**SVM**	An SVM model represents data samples as points in a space. The samples of separate categories are divided by a clear gap that should be as wide as possible. New data samples are then mapped onto that same space and predicted to become part of a category based on the side of the gap onto which they are mapped.	C=1.0, kernel='rbf', probability=True
**Neural Network**	Enter the Neural Network, a computational marvel inspired by the intricacies of the human brain's structure and function. Composed of interconnected nodes or neurons organized into layers, this model demonstrates a remarkable ability to learn intricate patterns	hidden_layer_sizes=(100, 100, 100, 100, 100), activation='relu',

	and representations from data. In the realm of machine learning, Neural Networks prove versatile for both classification and regression tasks. The deep learning subset, characterized by multiple hidden layers, empowers the model to grasp hierarchical features and complex relationships within the data.	random_state=11
**KNearest Neighbor**	The k-Nearest Neighbors (kNN) algorithm takes a unique approach, playing the role of a versatile non-parametric and instance-centric method applicable to both classification and regression scenarios. Grounded in the philosophy of proximity, kNN classifies a data point by assessing the majority class among its k closest neighbors in the feature space. Essentially, it operates under the assumption that instances sharing similar feature values are likely to belong to the same class. The parameter 'k' serves as a crucial tuning knob, determining the number of neighbors considered and, subsequently, the model's sensitivity to localized variations. Although kNN boasts an intuitive nature and simplicity, achieving optimal results demands a thoughtful selection of distance metrics and preprocessing techniques to address diverse feature scales effectively.	n_neighbors=5, weights='uniform'
**XG Boost**	XGBoost implements gradient boosting with decision trees as the underlying learners. Whereas random forest employs individual trees in parallel to solve the same problem, XGBoost builds individual trees sequentially. Each tree is trained to resolve the prediction error remaining following the prior tree and thereby improves prediction. This offers another approach to building more complex and accurate models with trees while controlling individual tree depth and complexity.	n_estimators=100, learning_rate=0.1, seed=11
**Decision Tree Classifier**	The Decision Tree Classifier stands as a non-linear model that tactically dissects the data through recursive splits based on features, making decisions in a step-by-step fashion. Each internal node signifies a decision tied to a specific feature, while each leaf node corresponds to a class label or regression output. Renowned for its capacity to capture intricate decision boundaries, decision trees also boast interpretability. Ensemble methods like Random Forest and AdaBoost leverage decision trees as foundational learners, amplifying predictive prowess and generalization.	criterion='gini', max_depth=None, random_state=11

Table S2. The top 10 DEMs and bottom 10 DEMs contributing positively and adversely affecting the discrimination process.

Top 10 Features[1] for		
LAA	SAO	CES
'hsa-miR-16-5p',<br>'hsa-miR-223-3p',<br>'hsa-miR-30d-5p',<br>'hsa-miR-22-3p',<br>'hsa-miR-486-5p',<br>'hsa-miR-30c-5p',<br>'hsa-miR-126-3p',<br>'hsa-miR-146a-5p',<br>'hsa-miR-451a',<br>'hsa-miR-122-5p',	'hsa-miR-100-5p',<br>'hsa-miR-24-3p',<br>'hsa-miR-103a-3p',<br>'hsa-miR-23a-3p',<br>'hsa-let-7b-5p',<br>'hsa-miR-146a-5p',<br>'hsa-miR-126-3p',<br>'hsa-miR-181a-5p',<br>'hsa-miR-425-5p',<br>'hsa-let-7f-5p',	"hsa-miR-320a-3p',<br>'hsa-let-7i-5p',<br>'hsa-miR-26b-5p',<br>'hsa-miR-3613-5p',<br>'hsa-miR-21-5p',<br>'hsa-miR-92a-3p',<br>'hsa-miR-143-3p',<br>'hsa-miR-223-3p',<br>'hsa-miR-150-5p',<br>'hsa-miR-25-3p',
Bottom 10 Features[2] for		
LAA	SAO	CES
'hsa-miR-21-5p',<br>'hsa-miR-199a-3p,<br>'hsa-miR-26b-5p',<br>'hsa-miR-191-5p',<br>'hsa-miR-92a-3p',<br>'hsa-miR-423-5p',<br>'hsa-miR-199b-3p',<br>'hsa-let-7i-5p',<br>'hsa-miR-103a-3p',<br>'hsa-miR-409-3p',	'hsa-miR-16-5p'<br>'hsa-miR-92a-3p',<br>'hsa-miR-486-5p',<br>'hsa-miR-21-5p',<br>'hsa-miR-191-5p',<br>'hsa-miR-22-3p',<br>'hsa-miR-223-3p',<br>'hsa-miR-126-3p',<br>'hsa-miR-143-3p',<br>'hsa-miR-26b-5p',	'hsa-miR-16-5p',<br>'hsa-miR-92a-3p',<br>'hsa-miR-423-5p',<br>'hsa-miR-486-5p',<br>'hsa-miR-199a-3p',<br>'hsa-miR-199b-3p',<br>'hsa-miR-22-3p',<br>'hsa-miR-223-3p',<br>'hsa-miR-103a-3p',<br>'hsa-miR-126-3p',

[1] Top features (Top 10 contribution features): Identifies the most important miRNA features for each class when using 1,251 selected features through logistic regression. By determining these key features, it highlights their significant contribution to class discrimination, sorted in descending order of influence (with the most influential feature appearing first).

[2] Bottom features (Top 10 determine features): Determines the most influential miRNA attributes for other classes utilizing logistic regression with 1,251 chosen features. This approach pinpoints the pivotal features, underscoring their crucial role in differentiating classes, and arranges them in descending order based on their impact (with the foremost influential attribute leading the list).

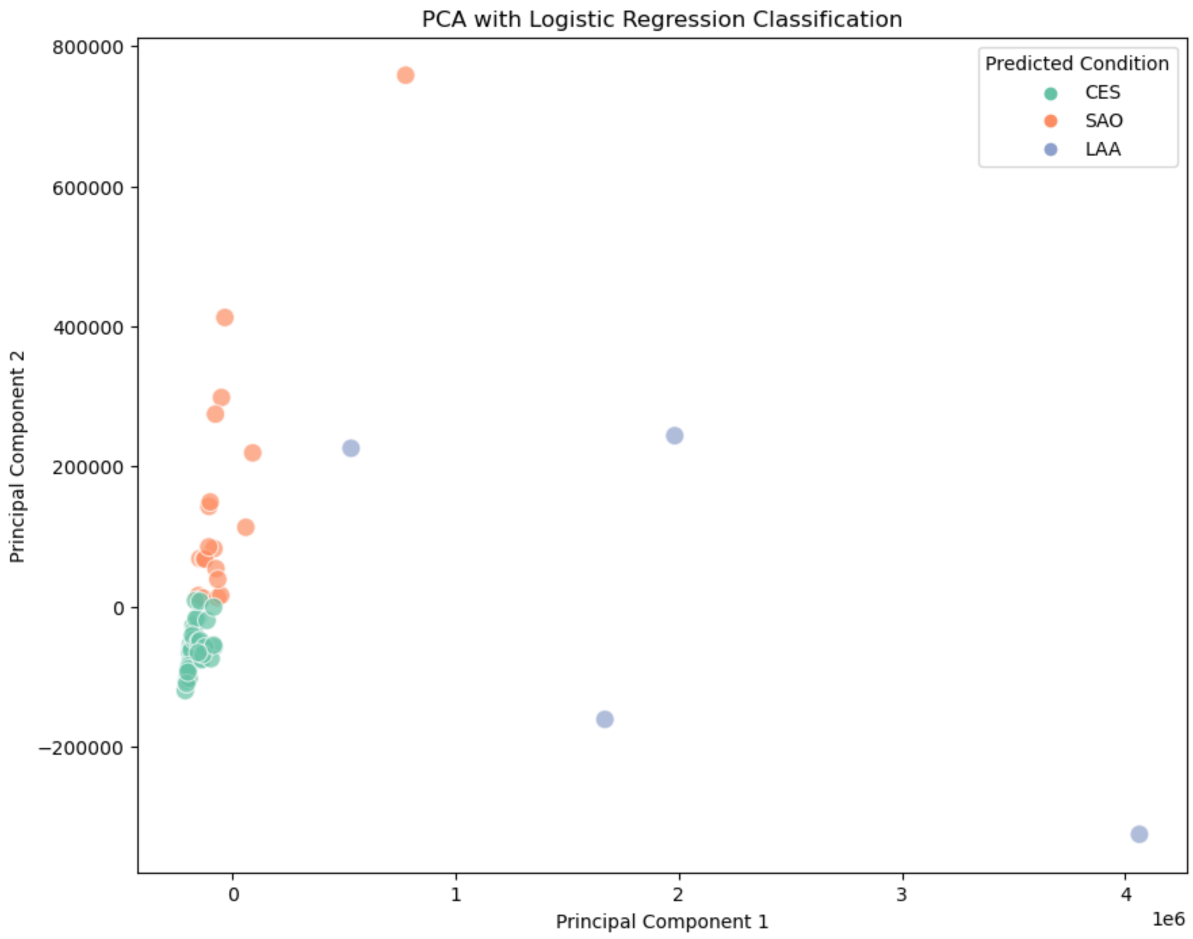

Figure S1. PCA accuracy for logistic regression**.** This scatter plot visualizes the results of a Principal Component Analysis (PCA) for dimensionality reduction, projected onto the first two principal components. The data points, colored based on three different conditions CES, SAO, and LAA show significant overlap, indicating that PCA, as an unsupervised learning technique, may not provide clear class separation even at its highest accuracy's component number which was 67. This suggests that, without considering labels in the data, PCA might have limitations in distinguishing between classes compared to supervised learning methods such as logistic regression, which directly incorporate class information to maximize predictive accuracy.

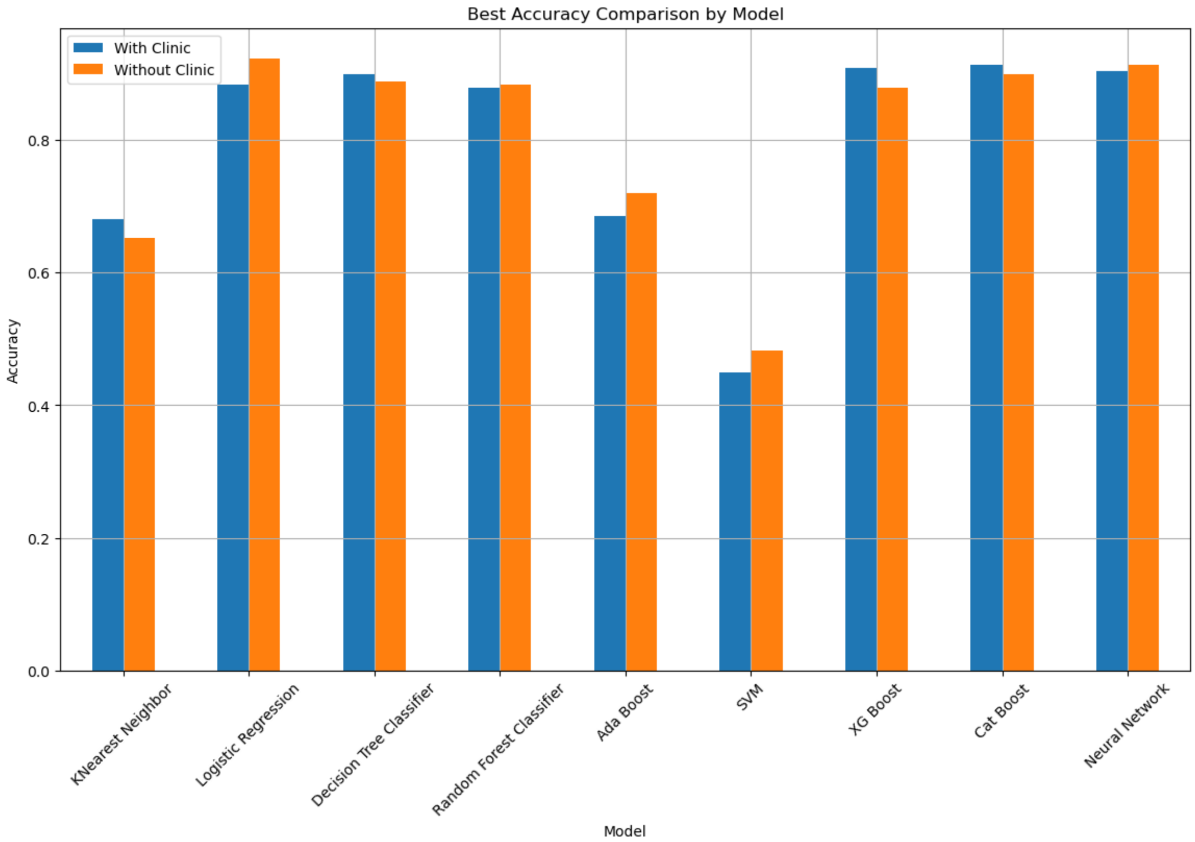

Figure S2: Machine Learning Model Performance for Stroke Subtype Classification with and without Clinical Information. We assessed the effect of combining clinical details (age, sex, and vascular risk factors) with miRNA expression profiles on stroke subtype classification accuracy. Using 3,024 miRNA features including clinical data ("With Clinic") and 3,017 features without it ("Without Clinic"), we analyzed various machine learning models. A bar chart illustrates that adding clinical information does not significantly change the accuracy of stroke subtype predictions. Surprisingly, the top-performing model did better without clinical data. This suggests that miRNA profiles alone might be sufficient for accurate stroke subtype diagnosis, encouraging further exploration into optimizing classification algorithms with less reliance on extensive clinical data.

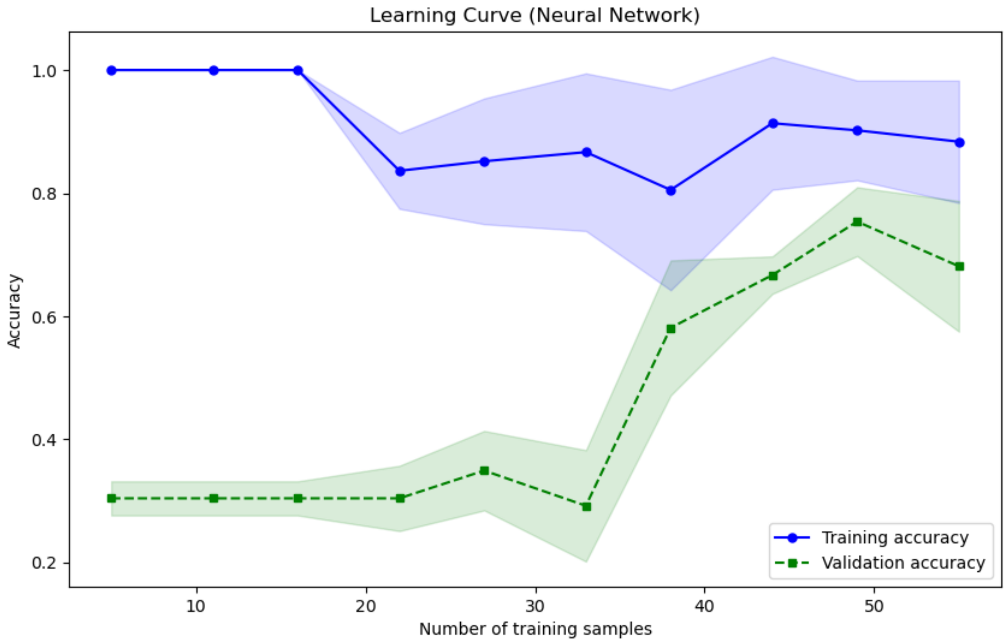

Figure S3: The learning curve analysis showed that more training samples would be necessary to improve the accuracy of neural network. In this study, the logistic regression had better accuracy compared to the neural network. However, this may be due to the neural network's characteristic of easily overfitting, especially when the sample size is small, leading to high training accuracy but lower validation accuracy. Neural networks tend to show an increase in validation accuracy as the amount of data increases (decreasing the generalization error), suggesting that with more data, neural networks could potentially outperform logistic regression. Therefore, it is expected that as we expand our dataset, the performance of the neural network will improve, surpassing logistic regression in handling complex pattern recognition tasks inherent to medical diagnostics.
